# The role of N-glycosylation modification in the pathogenesis of liver cancer

**DOI:** 10.1038/s41419-023-05733-z

**Published:** 2023-03-29

**Authors:** Mengyu Hu, Rui Zhang, Jiaren Yang, Chenshu Zhao, Wei Liu, Yuan Huang, Hao Lyu, Shuai Xiao, Dong Guo, Cefan Zhou, Jingfeng Tang

**Affiliations:** National “111” Center for Cellular Regulation and Molecular Pharmaceutics, Key Laboratory of Fermentation Engineering (Ministry of Education), Cooperative Innovation Center of Industrial Fermentation (Ministry of Education & Hubei Province), Hubei Key Laboratory of Industrial Microbiology, Hubei University of Technology, Wuhan, China

**Keywords:** Cancer microenvironment, Diseases

## Abstract

N-glycosylation is one of the most common types of protein modifications and it plays a vital role in normal physiological processes. However, aberrant N-glycan modifications are closely associated with the pathogenesis of diverse diseases, including processes such as malignant transformation and tumor progression. It is known that the N-glycan conformation of the associated glycoproteins is altered during different stages of hepatocarcinogenesis. Characterizing the heterogeneity and biological functions of glycans in liver cancer patients will facilitate a deeper understanding of the molecular mechanisms of liver injury and hepatocarcinogenesis. In this article, we review the role of N-glycosylation in hepatocarcinogenesis, focusing on epithelial-mesenchymal transition, extracellular matrix changes, and tumor microenvironment formation. We highlight the role of N-glycosylation in the pathogenesis of liver cancer and its potential applications in the treatment or diagnosis of liver cancer.

## Facts

- N-glycosylation modifications mediate multiple biological functions such as cell recognition, signal transduction, and immune response.
- The glycosylation pattern of tumor cells is often altered to facilitate cancer progression.
- The N-glycan conformation of related proteins may change to different degrees during the pathogenesis of liver cancer.

## Open questions

- What physiological processes are involved in the pathogenesis of liver cancer?
- What role does N-glycosylation play in the pathogenesis of liver cancer?
- Does N-glycosylation modification have practical application prospects in the diagnosis and treatment of liver cancer?

## Introduction

Liver cancer is the fifth most common cancer type in the world, and the second leading cause of cancer-related death, with more than 900,000 new cases and 830,000 deaths annually [1]. Liver cancer is a great threat to human health, especially among Asian people.

Primary liver cancer accounts for the largest proportion of liver cancer cases. The liver cancer involved in this review refers to primary liver cancer. According to different pathological features, primary liver cancer can be classified into hepatocellular carcinoma (HCC), intrahepatic cholangiocarcinoma, and the extremely uncommon combined hepatocellular cholangiocarcinoma [2]. Approximately 90% of these cases of conventional liver cancer are attributed to HCC [3]. HCC can develop in response to any cirrhosis risk factor, such as hepatitis B virus (HBV) infection, hepatitis C virus (HCV) infection, non-alcoholic steatohepatitis, aflatoxin exposure, obesity, and diabetes mellitus [4]. Liver cancer is aggressive, exhibiting rapid cell growth, effective angiogenesis, and a prominent anti-apoptosis phenotype—all driven by pathogenic mechanisms that remain unclear [5].

**Fig. 1:**
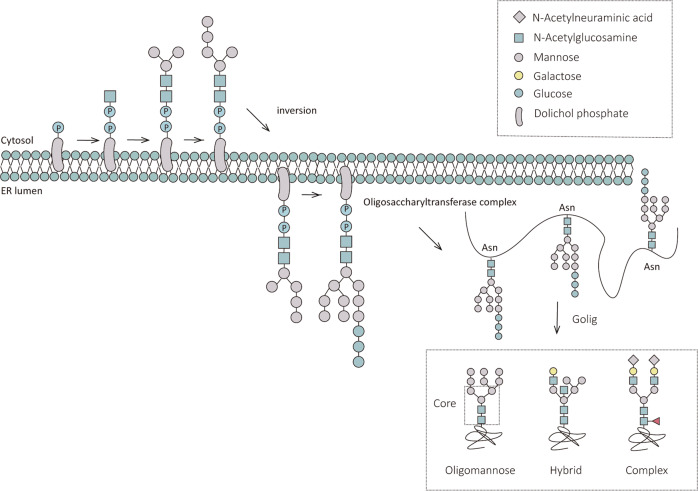
The process of N-glycosylation. N-glycosylation modifications begin at the endoplasmic reticulum and end at the Golgi apparatus. The new oligosaccharide chains are initially processed in the endoplasmic reticulum and then transferred to the N-glycosylation sites of the new peptide chains by the action of the oligosaccharyltransferase complex (OST), and the attached polypeptide chains will undergo further processing and modification in the Golgi apparatus to form three N-glycan types, oligomannose, hybrid, and complex.

Glycosylation affects the biological functions of normal cells. It plays a vital role in the pathogenesis of cancer and is one of the most common types of post-translational protein modification [6]. Glycosylation modification can be classified as N-glycosylation and O-glycosylation based on the different types of amino acid residues linked with the glycan chain. N-glycosylation occurs at the amino acid sequence NXS/T(X ≠ P), and O-glycosylation occurs at the S/T site [7]. Glycosylated proteins, N-linked glycosylated proteins in particular, are predominant among proteins destined for extracellular environments. Here we focus on N-glycosylation modifications. First, Dolichol phosphate binds to the glycan chains to form the glycolipids Dolichol-linked oligosaccharides (DLOs), which serve as glycan donors to provide substrates for N-glycosylation [8]. The mature DLOs consist of two N-acetylglucosamines, nine mannoses and three glucoses. Subsequently, the N-glycan is recognized by the oligosaccharyltransferase (OST) complex, and the OST complex mediates its transfer to the N site on the nascent polypeptide chain that harbors an NXS/T(X ≠ P) sequence combination [7]. Finally, the N-glycan of the bound polypeptide chain will enter the Golgi for further processing and modification to form three different N-glycan classes: oligomannose, hybrid and complex [9]. The abovementioned is the main process of N-glycosylation. A schematic diagram is shown in Fig. 1.

A slight change in the amount of glycosyltransferase or glycan donor in the body can lead to a great variation in the conformation of polysaccharides [10]; this is closely related to a massive change in life activities [11]. The association between glycoprotein levels and conformational variations and cancer development has been recognized for a long time. As early as 1978, Rostenberg et al. found statistically significant differences in alpha1-antitrypsin (alpha1-AT) glycosylation levels between patients with lung, prostate, and gastrointestinal cancers and control patients with benign tumors [12]. Although the authors did not conduct further studies on the differential glycoforms of alpha1-AT, their results clearly indicate that the glycosylation pattern of alpha1-AT is changed in different cancer types. In the 1990s, Hakomori systematically summarized the impact of abnormal glycosylation on tumorigenesis and tumor development, providing a framework for subsequent researchers [13]. During this period, studies on the connection between N-glycosylation status and the advancement of cancer have increased.

**Fig. 2:**
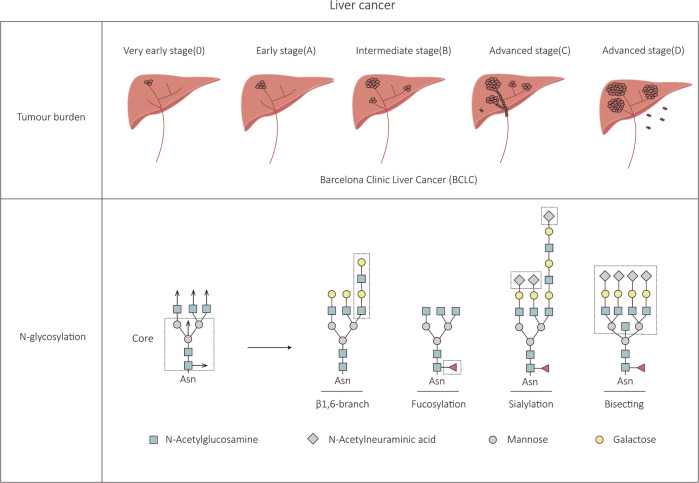
Schematic diagram of liver cancer progression and associated N-glycan type changes. Barcelona Clinic Liver Cancer (BCLC) is the most widely used clinical liver cancer staging system, which divides liver cancer into three stages, early (0/A), intermediate (B), and advanced (C/D), mainly based on the patient’s tumor status and liver function. N-glycan chains can participate in cell recognition, signal transduction, immune responses, and other life activities. Compared with normal cells, tumor cells, such as HCC cells, often exhibit abnormal changes in N-glycan conformation, such as abnormal branching chains and bisecting structures and altered levels of fucosylation and sialylation.

Liver cancer, one of the most prevalent malignancies, has also been a popular research subject. Certain N-glycosylation changes have been associated with liver cancer in particular. It is difficult to detect early-stage liver cancer because of a lack of reliable markers. The first study on the N-glycan chain categories of serum glycoproteins in patients with liver cancer was conducted by Turner in 1992, whose results showed that the levels of sialylation and fucosylation of N-glycan chains were strongly increased when liver lesions progressively developed into liver cancer [14] (Fig. 2). DelaCourt et al. further confirmed that there is a correlation between HCC genotype and N-glycosylation heterogeneity [15]. This suggests that targeting aberrant glycosylation modifications may facilitate disease diagnosis. Later research revealed that the N-glycosylation of envelope proteins is also necessary for the secretion of hepatitis virus particles [16, 17]. Jiang et al. discovered that the N-glycan chain on Golgi Protein 73 (GP73) Asn144 reduces the invasive ability of HCC cells by mediating intercellular adhesion [18]. Recently, it has been demonstrated that N-glycosylation of residues 294 and 454 of the Mer tyrosine kinase (MerTK), a crucial factor in the prognosis of HCC, can promote the proliferation and transformation of HCC cells [19]. It is clear that N-glycosylation alterations change the outcome of liver cancer development. The interest in investigating the glycosylation status of N-glycoproteins has remarkably increased in recent years due to its potential value for early diagnosis of liver cancer and effective therapy.

The pathogenesis of liver cancer involves biological processes related to epithelial-mesenchymal transition (EMT), extracellular matrix (ECM) changes, and tumor microenvironment (TME) formation [20]. The association between N-glycosylation upon HBV infection and during hepatocarcinogenesis has been generally described previously [21]. In this review, we discuss the physiological functions of N-glycosylation of related proteins in the abovementioned biological processes and propose potential strategies targeting N-glycosylation in the diagnosis and treatment of liver cancer.

### Effect of N-glycosylation modification on epithelial-mesenchymal transition

Epithelial-mesenchymal transition (EMT) occurs when cells lose their epithelial properties and acquire mesenchymal properties which contribute to tumor progression [22].

The established notion is that EMT is correlated with tumor aggressiveness and poor outcome. A large amount of experimental evidence has shown that a variety of effectors are involved in the process of EMT in the development of HCC to promote the occurrence and pathogenesis of HCC [23]. E-cadherin, N-cadherin, Epithelial Cell Adhesion Molecule (EpCAM), vimentin, and other marker proteins that are frequently employed to identify the EMT process are crucial in stimulating normal liver cells to develop mesenchymal traits [24]. The investigators selected 145 clinical cases related to liver cancer, of which about 50% of cirrhotic patients were caused by HCV infection, and analyzed the expression levels of EMT-associated proteins in serum samples from patients in the normal, cirrhotic, and HCC groups. Comparing HCC patients to cirrhotic and healthy individuals, HCC patients showed the lowest levels of E-cadherin [25]. Moreover, deletion or downregulation of E-cadherin greatly promoted the invasive metastatic phenotype of liver cancer cells, conducive to the EMT process [26].

In HCC, related transcription factors such as Snail and Twist are often upregulated to accelerate the development of EMT [27]. In addition, some signaling pathways, such as the Wnt/β-catenin pathways that drive EMT, are also activated [28]. Under normal physiological conditions, β-catenin can bind to the intracellular structural domain of E-cadherin to enhance intercellular adhesion [29]. However, during the EMT process, the interaction between them is weakened; β-catenin dissociates from the calmodulin complex and is translocated to the nucleus, where it plays a regulatory role, such as increasing the expression levels of Snail and ZEB1 in HCC cells, which results in weakened cell adhesion and increased motility [30].

**Fig. 3:**
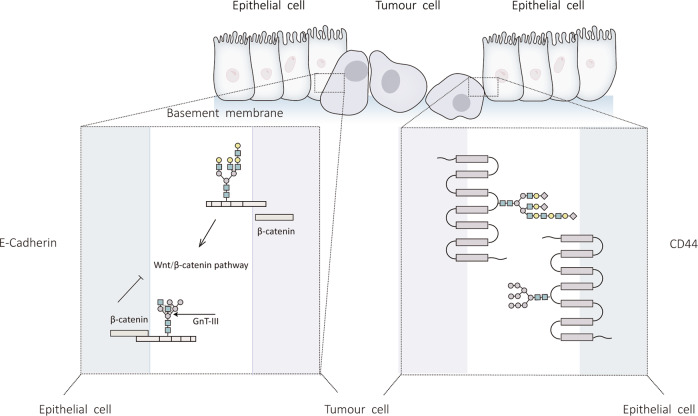
Effect of N-glycosylation modification on EMT. A large body of experimental evidence shows that EMT is an indispensable part of the occurrence and development of liver cancer, which involves the abnormal N-glycosylation of related proteins. This diagram summarizes the abnormal N-glycosylation of associated proteins during EMT in liver cancer.

Therefore, EMT is a crucial process in the development of liver cancer. As previously discussed, tumor cells typically have varying degrees of glycosylation. In the following, we will elaborate on the influence of N-glycosylation modification of related proteins on the process of EMT in liver cancer (Fig. 3).

#### E-cadherin

The N-glycan chain conformation of E-cadherin, a marker protein of epithelial cells and a transmembrane glycoprotein, greatly affects its adhesive properties [31], and in malignant tumors the N-glycan chains of E-cadherin tend to form a more complex branching chain structure [32], which promotes internalization of E-cadherin into the cytoplasm and disrupts intercellular adhesion, thereby disrupting the relevant signaling pathways and facilitating the occurrence of EMT [33].

As previously mentioned, β-catenin enters the nucleus following its dissociation from E-cadherin to activate the Wnt signaling pathway and promote the expression of target genes for EMT in HCC cells [30]. In addition, the N-glycan chain of E-cadherin controls the contact between E-cadherin and β-catenin. According to the findings of Xu et al., N-acetylglucosaminyltransferase 3 (GnT-III) is involved in this process [34]. GnT-III catalyzes the production of β-1,4 glycosidic linkages, resulting in the formation of the bisecting chain of E-cadherin. The results of Xu et al. further indicate that when TGF-β1 promotes EMT, the expression of GnT-III is downregulated, leading to a reduction of the bisecting N-glycan structure formed by E-cadherin and inhibition of the interplay between E-cadherin and β-catenin [35]. Then β-catenin stimulates the Wnt signaling pathway by nuclear translocation, accelerating EMT. The abovementioned conclusion clarified the value of the N-glycan structure of E-cadherin in EMT for the first time. Mo et al. confirmed this opinion in MHCC97-L cells, indicating that the mechanism may also be applicable to liver cancer [36].

This implies that important enzymes mediating the process of N-glycosylation modification, such as GnT-III, can be used as potential targets for liver cancer therapy.

#### EpCAM

Epithelial cell adhesion molecule (EpCAM) is a type I transmembrane glycoprotein that was once thought to be a tumor-associated antigen due to its high expression in quickly growing epithelial malignancies [37]. EpCAM has three N-glycosylation sites, which is Asn77, Asn111, and Asn198 [38]. It has been reported that when all the above sites are mutated, the half-life of EpCAM decreases from 21 to 7 h [39], which suggests that N-glycosylation can stabilize the protein.

EpCAM is present in a variety of cancer types and is crucial for tumor metastasis, cell proliferation, and adhesion. As one of the most important post-translational modification types, N-glycosylation is also involved in the abovementioned activities. There have been claims that when the N-glycan chain of EpCAM is disrupted in breast cancer cells, the expression of pro-apoptotic proteins Bax and Caspase 3 is increased, showing that EpCAM hinders apoptosis through its N-glycan [40]. Moreover, Liu et al. counseled that the N-glycan of EpCAM could enhance the adhesion of breast cancer cells [41]. When N-glycosylation sites were altered, the interaction between EpCAM and fibronectin (FN) was compromised and intercellular adhesion was decreased. Thus, N-glycosylation of EpCAM has extremely important physiological effects in breast cancer, which may also apply to other cancer types such as liver cancer.

In human liver cancer samples, EpCAM is often co-expressed with EMT marker proteins [42] and cancer stem cell biomarkers [43]. EpCAM-positive HCC cells are highly invasive [44]. Sancho-Bru et al. reported that EpCAM is considerably upregulated in patients with alcoholic hepatitis compared with the normal group [45]. When EpCAM was knocked down, the PI3K/Akt/mTOR signaling pathway was arrested, and the level of liver fibrosis in alcoholic hepatitis mice was improved [46]. These results indicate that EpCAM may serve as a target for liver cancer treatment. Based on the function of the N-glycan chain of EpCAM in breast cancer cells, it is reasonable to speculate that targeting the N-glycosylation of EpCAM may also have unexpected effects in liver cancer. More experimental evidence is needed to validate this conjecture.

#### CD44

Lymphocyte homing receptor (CD44) is a non-kinase transmembrane glycoprotein that is expressed in both embryonic stem cells and connective tissue [47]. CD44, as one of the common tumor stem cell biomarker, can regulate EMT and participate in the progression of tumors [48].

CD44 has a globular N-terminal hyaluronic acid binding domain (HABD), which binds specifically to hyaluronic acid [49]. This binding alters the conformation of CD44, activating various intracellular signaling pathways and regulating cell biological behaviors [50]. CD44 has been shown to participate in EMT by binding to its ligand hyaluronic acid [51]. The role of N-glycosylation in this process will be further elaborated below.

Specific residues on glycoproteins are important for recognition of other biomolecules. Changes in the glycan composition of glycoproteins greatly affect these interactions, such as the interaction between CD44 and hyaluronic acid. Their binding is highly regulated by glycosylation of the HABD [52]. Multiple N-glycosylation sites exist in the HABD of CD44 [53]. The interaction between CD44 and hyaluronic acid is highly influenced by the N-glycan conformation of HABD [54]. The complex N-glycan structure on HABD reduces the recognition ability between them. In addition, when N-glycan on the HABD is acidified by saliva, this also inhibits the binding of CD44 to its ligand, thus affecting the function of CD44 [55]. Studies have shown that the binding activity between CD44 and hyaluronic acid is notably enhanced after sialic acid molecules of the N-glycosylation chain on CD44 are specifically removed by treating HCC cells with sialidase NEU4, which inhibits the migration of HCC cells [56]. These results provide a new feasible idea for controlling HCC cell motility and metastasis.

### Effect of N-glycosylation modification on the extracellular matrix

In terms of spatial distribution, the extracellular matrix (ECM) consists of two major components, the basement membrane, and the interstitial matrix, and the ECM is rich in fibronectin (FN), proteoglycans, and stromal cell proteins [57]. Under normal physiological conditions, these components are combined in a specific way to provide a suitable mechanical environment for cells, giving the matrix unique physical and biochemical characteristics, affecting cell morphology, metabolism, and migration [58].

In reality, the ECM is in a state of orderly reconstruction in normal organisms, and its components are constantly circulating in the three processes of secretion, modification, and degradation to adapt to and maintain tissue homeostasis [59]. However, during cancer development, this process is dysregulated, and although the ECM can act as a physical barrier to prevent tumor cell proliferation and invasion in the early stages, once tumor cells break through the basement membrane, prompting changes in the composition and structural properties of the ECM, the tumor stroma formed by ECM remodeling will facilitate tumor cell growth, survival, and invasion [60].

The liver has a pronounced capacity for regeneration in its role as a metabolite-detoxifying organ. This process generally starts with the activation of associated enzymes, such as MMPs, for the degradation of abnormal ECM components [61], followed by the stimulation of growth factors, such as TGF-β, to start proliferative remodeling [62]. Compared with other solid tumors, the degree of fibrosis in the liver is high after organic lesions, and excessive fibrosis can lead to cirrhosis, which eventually develops into HCC if not treated properly. Pathological damage to the liver occurs in response to various pathogenic factors and is essentially caused by excessive accumulation of ECM proteins [63]. In chronic liver injury, although the damaged liver will regenerate and rebuild, the new liver tissue no longer has its original structural features and functional characteristics [64]. The formation of aggregated fibrous bundles of ECM components that are difficult to degrade during the repair and reconstruction of multiple injuries, known as fibrous scars, which can obstruct blood flow of liver tissues and are the cause of liver tissue lesions, is the most common adverse effect of this repair method used to maintain homeostasis of the internal environment in chronic liver injury [65].

**Fig. 4:**
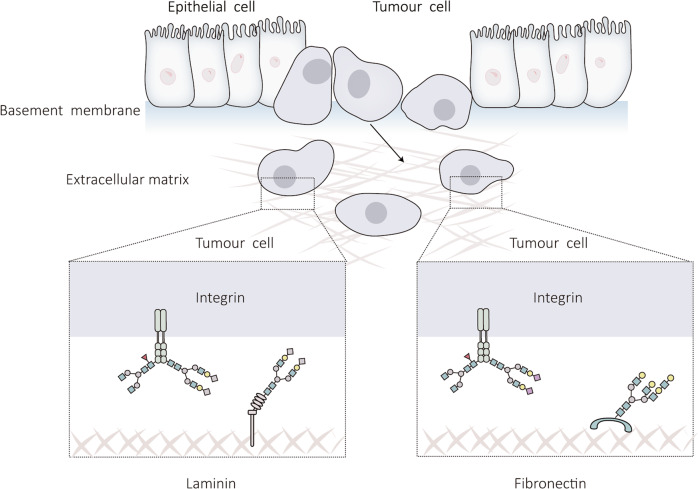
Effect of N-glycosylation on the ECM. Under normal physiological conditions, the components of the ECM can provide a suitable mechanical environment for cells. However, in liver cancer, the ECM is disordered, which is closely related to the N-glycosylation of ECM components. This figure outlines the effect of N-glycosylation on ECM homeostasis in liver cancer.

ECM dysregulation is associated with abnormal expression of its components [66], and the role of N-glycosylation modifications in this process will be discussed below (Fig. 4).

#### Integrin

Integrins are transmembrane glycoprotein receptors that bind to ECM components and trigger signaling cascades to regulate cellular homeostasis and normal developmental processes in the organism [67]. Integrins are composed of an α subunit and a β subunit. At present, 24 different integrin categories have been identified in vertebrates, with different ligand binding properties, serving as a bridge for information transmission between cells and the ECM [68]. For example, α5β1 integrin can recognize the Arg-Gly-Asp tripeptide sequence in FN and regulate intercellular adhesion [69], while α3β1 integrin can recognize its ligand-linked adhesion molecule (JAM-A) and promote the migration of polarized epithelial cells [70].

Abnormal glycosylation modifications, including sialylation and core fucosylation, are common in tumor cells and play a conspicuous role in cancer progression [71]. As the main N-glycan carrier, integrin has more than 20 potential glycosylation sites, and the core structure of N-glycan is crucial for the formation and function of heterodimers [72]. Han et al. revealed that miRNA can be employed as a tumor suppressor by regulating the sialic acid content of integrin 1 via the sialic acid transferase ST6Gal-I, preventing HCC cells from spreading [73]. In addition, it has been found that inhibition of the core fucosylation of integrin in HepG2 cells affects its downstream signaling pathway, resulting in significant suppression of cell proliferation and migration [74]. The abovementioned studies have shown that the abnormal N-glycan modification of the integrin subunit can affect its affinity to ligands, regulating cell function and making it tend to the malignant phenotype, suggesting that abnormal sialylation and fucosylation on targeted integrin N-glycan can be used as a potential way for HCC treatment.

#### Fibronectin

Fibronectin (FN) is a glycoprotein with a large molecular weight that is involved in wound healing and embryo development. It consists of two subunits, each of which is made up of three repeat modules with identical protein folding conformations, such as types I, II, and III [75]. Due to the selective splicing of mRNA, FN has different structural characteristics, which affects its solubility. Soluble FN exists in plasma and regulates cell adhesion, promoting wound healing, while insoluble FN exists in the ECM, mediating cell growth and differentiation [76].

Hsiao et al. reported the plasma FN N-glycan site and discovered that the N-glycan conformation of plasma FN influences its interaction with integrins, altering cell adhesion [77]. Therefore, N-glycan conformational changes could regulate the functional properties of plasma FN. Subsequently, Liu et al. analyzed the glycan types of human plasma FN, Mass spectrometry (MS) analysis revealed that 77.31 % of the six identified N-glycosides were sialic acidified [78], enriching our understanding of the site-specific glycosylation patterns of FN. Vascular infiltration is one of the main risk factors for recurrence and poor prognosis of liver cancer patients. It has been reported that FN is a biomarker of invasive HCC [79]. Linking FN with its N-glycosylation is helpful to find new therapeutic targets and improve the condition of patients.

#### Laminin

Laminin is an ECM glycoprotein found in the basement membrane that promotes cell adhesion and migration, maintains tissue structural stability and mediates specific physiological functions through interactions with cell surface receptors and stimulation of intracellular signaling cascade responses [80]. Laminins are heterogeneous trimers composed of five α-chains, four β-chains, and three γ-chains, but of the 60 possible heterogeneous trimers, only 16 have been confirmed biochemically [81]. Ln-332 is composed of α3, β3, and γ2 chains and mediates the adhesion between epithelial cells and the basement membrane. Ln-γ2, a component of Ln-332, is often expressed as a monomer in malignant tissues, and additionally, Ln-γ2 monomers can induce tumor cell proliferation and migration in vitro [82], suggesting that Ln-γ2 monomer can be used as a potential cancer detection marker to facilitate the screening of cancer and improve diagnosis. Yasuda et al. found that with the combined application of Ln-γ2 monomer and vitamin K deficiency-induced prothrombin (PIVKA-II) in clinical HCC detection, the diagnosis may be more reliable [83]. In addition, Ln-332 is a large heterogeneous trimeric glycoprotein, and relatively little is known about the functional impact of N-glycosylation on this class of proteins. Kariya et al. showed that cell adhesion was decreased when overexpression of N-acetylglucosaminyltransferase III (GnT-III), induced the formation of more diastereomeric N-glycan structures; however, intercellular adhesion was remarkably enhanced when overexpression of N-acetylglucosaminyltransferase V (GnT-V), induced the formation of more β1,6-branched N-glycan structures [84]. The abovementioned results demonstrated that N-glycosylation could regulate the biological function of Ln-332. This finding may lead to new therapeutic strategies for liver cancer.

### Effect of N-glycosylation modification on tumor microenvironment

The tumor microenvironment (TME) is a dynamic system that surrounds a tumor inside the body that is widely implicated in tumorigenesis [85]. This system contains a variety of resident and infiltrating host cells, secreted factors, and ECM proteins [86]. Emerging evidence shows that TME-mediated changes in glycosylation play an important functional role in tumorigenesis.

The TME consists of a large number of immune cells. These immune cells can be split into two main types, tumor-antagonizing immune cells and tumor-promoting immune cells, based on their impact on the development of cancer. Tumor-antagonizing immune cells, such as CD8^+^ cytotoxic T cells, target and kill tumor cells in the early stages of cancer; tumor-promoting immune cells, such as Myeloid-Derived Suppressor Cells (MDSCs), suppress the immune response in the body and support cancer progression [87].

As an immune regulatory organ, the liver contains a variety of immune cells, such as natural killer cells and dendritic cells (DCs), which can mediate a series of immune response to eliminate pathogens and sustain internal environmental homeostasis [88]. The immune system of the body is out of balance in liver cancer, which causes excessive release of pro-inflammatory factors in the TME [89]. This causes a sustained inflammatory response in the liver, pushing the healthy liver in the direction of fibrosis and cirrhosis and ultimately leading to the development of liver cancer [90].

N-glycosylation is crucial for regulating immune cell differentiation and maturation [91]. DCs are the most powerful antigen presenting cells (APCs), mediating innate immunity and inducing adaptive immunity. Studies have revealed that sialylation levels drastically decrease during the maturation of DCs [92]. Further analysis unveiled that when mouse bone marrow-derived DCs (BMDCs) were treated with sialic acid blocking mimics, the viability and differentiation degree of BMDCs were not affected, but their maturation was obviously boosted, and the proliferation levels of BMDCs-induced antigen-specific CD8^+^ T cells were raised, impeding the progression of cancer development [93]. After stable overexpression of β-Galactoside α2-6-sialyltransferase 1 (ST6Gal-I) in HepG2 cells, the number of CD8^+^ T cells infiltrated in the TME was reduced, so the content of IFN-γ and TNF-α secreted by them were decreased, which is beneficial to liver cancer progression [94].

The impact of N-glycosylation on immune cells in the TME of liver cancer will be discussed below (Table 1).

**Table 1:**
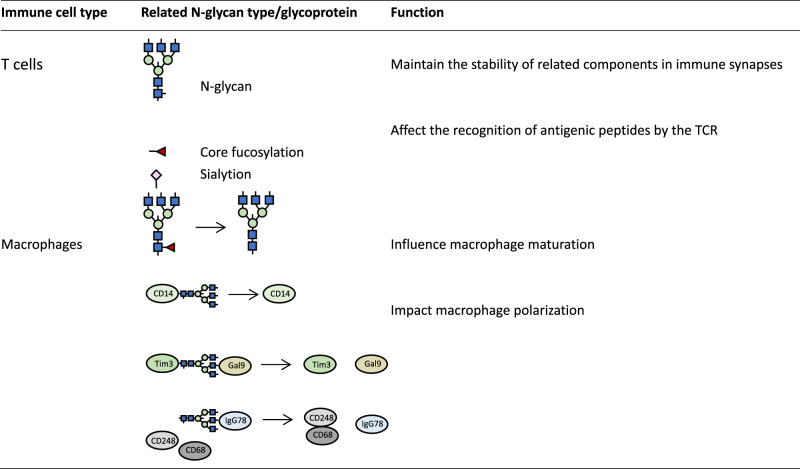
Effect of N-glycosylation on immune cells in the TME of liver cancer.

N-Acetylneuraminic acid  N-Acetylglucosamine  Mannose

#### T cells

T cells are present in almost every organ and tissue of the body, including the liver. T cells are functionally diverse lymphocytes differentiated from hematopoietic stem cells in the bone marrow, which develop and mature in the thymus [95]. In the early stages of liver cancer, T cells are activated after receiving antigen-presenting signals from APCs, after which they infiltrate into the TME and fight against liver cancer cells, while the glycosylation patterns displayed by T cells are directly related to their maturation and activation [96].

As one of the crucial elements of T cell recognition of antigens, T cell receptor (TCR) plays a key role in T cell activation. This process involves the formation of immune synapses, where the TCR-antigenic peptide-major histocompatibility complex (MHC) forms the central region of the immune synapse, and the interaction of the abovementioned components stimulates the immune function of T cells [97]. The current findings suggest that the N-glycan chain on the TCR and MHC maintains the stability of related components in immune synapses, which inhibits their degradation by the proteasome [98, 99].

Tyrosinase is a common tumor antigen. When the N-glycan chain at its Asn317 site was disrupted, its presentation by MHC-I was greatly increased [100]. The recognition of antigenic peptides by TCR was much improved once the N-glycan chain was eliminated, which aided the ensuing immune response [100]. In addition, the antigenicity and immunogenicity of the hepatitis B surface antigen (HBsAg) are compromised with the attachment of extra N-glycan [101]. In this way, HBV molecules generate new N-glycan branches on HBsAg to obscure the original antigenic epitopes recognized by the TCR for immune escape [102]. This indicates that the N-glycan chain affects the recognition of antigenic peptides by the TCR. However, terminal N-glycan modifications on antigenic peptides or TCRs, such as fucosylation, are involved in the activation of T cells [103]. Sialylation is one of the classes of terminal N-glycosylated modifications and is essential for the regulation of immune function. According to a study by Oswald et al. [104], liver tissue exhibits a strong inflammatory response when 2,6-sialic acid branched chains are specifically removed from the surface of mouse liver cells. The T cell-mediated immune response is also triggered. This conclusion can be supported by the experimental data of Liu et al. [105]. The effect of N-glycosylation on the immune response is also correlated with terminal N-glycan modification.

Clarifying the aberrant N-glycosylation pattern of immune cells in the TME of liver cancer will help us better understand the pathogenesis of this cancer type.

#### Macrophages

Similar to T cells, macrophages are distributed throughout the body, but macrophages mediate the innate immune response. The majority of hepatic macrophages are Kupffer cells. When liver tissue is damaged, peritoneal and bone marrow-derived macrophages migrate to the liver, working with Kupffer cells and other immune cells to counteract the effects of the injury [106].

Based on their functional characteristics, macrophages can be divided into M1 and M2 macrophages. M1 macrophages positively regulate the immune response, while M2 macrophages secrete inflammatory inhibitory factors such as TGF-β, negatively controlling the immune response [107]. In liver cancer, the proportions of M1/M2 macrophages are always fluctuating, promoting or inhibiting TME formation [108]. Targeting macrophage subtypes to reverse the inflammatory response is a promising strategy for liver cancer treatment. During the differentiation and maturation of macrophages, their surface N-glycans undergo remodeling [109]. Yang et al. investigated the classes of N-glycans of mouse peritoneal macrophages and identified 587 N-glycoprotein components [110]. Their data showed that the N-glycosylation pattern of these proteins was notably remodeled during macrophage activation, and the level of core fucosylation expressed by mature macrophages was remarkably reduced [110]. CD14 is a macrophage marker protein, and its N-glycan depletion disrupts its membrane localization, thus affecting the polarization process of macrophages [110].

Galactose lectins are a class of proteins with a specific affinity for β-galactoside, which is a fundamental regulator of cell microenvironment remodeling, and its aberrant expression has been linked to a variety of clinical diseases [111, 112]. It has been reported that abnormal expression of Gal-1 and Gal-3 is closely related to the invasion and migration of liver cancer cells and poor prognosis of liver cancer patients [113, 114]. Gal9 mediates macrophage polarization as a ligand for N-glycosylated T-cell immunoglobulin mucin 3 (Tim-3) [115], which may lead to liver cancer cell death [116]. CD248 is a C-type lectin-like receptor, which is mostly expressed in cancer tissues and can bind to glycoprotein components in the TME to participate in related inflammatory reactions [117]. In HCC, CD248 is expressed on CAFs and interacts with the macrophage marker protein CD68, promoting macrophage M2 polarization [118]. IgG78 is a specific antibody targeting glycosylated CD248, which reduces the expression level of CD248 in CAFs and decreases the production of M2 macrophages, exerting anti-tumor effects [118]. While the targeting effect of IgG78 on CD248 is highly correlated with the level of N-glycan on CD248, the anti-tumor effect of IgG78 is diminished when the N-glycan chain of CD248 is disrupted [118].

The interaction between N-glycan proteins and their ligands or receptors tremendously influences the polarization of macrophages, and targeting the N-glycosylation regulatory mechanisms of the corresponding proteins would help to alleviate the inflammatory response in the liver.

### Application of N-glycosylation in liver cancer diagnosis

The treatment of patients with HCC is closely related to their clinical diagnosis. Microwave ablation is typically used to treat early-stage patients; liver transplantation and hepatic resection are typically used to treat mid-stage patients; while advanced patients can only be treated with clinical systemic pharmacological treatments to slow down the disease process, commonly first-line drugs such as sorafenib [92].

For patients with early liver cancer, surgical treatment is the first choice, but for patients who need liver transplantation, it is not easy to find suitable liver donors [119]. Although drug therapy can improve the overall survival rate of advanced patients, the side effects of this treatment cannot be ignored [120]. This kind of risk could be avoided by techniques such as early screening. The establishment of early screening indicators could reduce the risk of death to a great extent, and improve patient survival. However, screening for liver cancer is not an easy task. Most patients are diagnosed in the middle to late stages and miss the best time for treatment, so it is important to establish accurate and reliable screening indicators [121].

Cancer development and N-glycosylation are tightly connected. Some N-glycosylated proteins are also common indicators for the diagnosis of liver cancer. Below, we will list the application of N-glycosylation of some proteins in the diagnosis of liver cancer (Box 1, Table 2).

**Table 2:** Important N-glycosylation events in liver cancer.

Protein	N-glycosylation events in liver cancer	Outcome/Application
E-Cadherin	Reduce bisecting N-glycan structure	The interaction between E-Cadherin and β-catenin is weakened, and β-catenin enters the nucleus to activate the Wnt signaling pathway and promote the EMT process
CD44	Increase sialylation levels	Promote the intercellular adhesion
Integrin	Raise sialylation and core fucosylation levels	Inhibit the proliferation of liver cancer cells
Fibronectin	Elevate sialylation levels	Influence its interaction with integrins, altering cell adhesion
Laminin	Increase the formation of β1, 6-branch	Enhance intercellular adhesion.
AFP	Expand core fucosylation levels	More reliable clinical diagnostic and prognostic indicator of liver cancer
AGP	Raise sialylation levels	Increase diagnostic accuracy of liver cancer
Haptoglobin	Widen sialylation levels, lessen the formation of N-glycan branching structure	Reduce false negative results of diagnosis

N-Acetylneuraminic acid  N-Acetylglucosamine  Mannose  Galactose

Box 1 Application of N-glycosylation in liver cancer diagnosis ***AFP***

AFP, a common liver cancer diagnostic indicator, is not sufficient by itself to determine whether a patient is diagnosed, but needs to be combined with Computed Tomography and other techniques [128**]**. Although most patients with liver cancer have high serum AFP levels, liver cancer is not the only cause of elevated AFP, as abnormalities in this indicator can also be detected in patients with lung cancer [129] or pancreatic cancer [130] and in pregnant women.

It has been established that abnormal glycosylation is a crucial component of carcinogenesis and cancer development, including HCC. We mentioned earlier that AFP is an N-glycosylation-modified protein with multiple N-glycosylation sites [131]. AFP can be classified into three groups, AFP-L1, AFP-L2, and AFP-L3, depending on how well they bind to the leptin Lens Culinaris Agglutinin (LCA). AFP-L1 is the main AFP type and does not bind specifically to LCA; AFP-L2 can be found in pregnant women and has an average binding ability to LCA; AFP-L3 has the strongest binding ability to LCA [132]. Core fucosylation at site 251 of AFP-L3 have been reported, and such abnormal N-glycosylation modifications are closely associated with malignant disease progression [133]. Serum AFP-L3 has also been used as a clinical diagnostic and prognostic indicator for the prevention and treatment of liver cancer, which is more reliable.

***AGP***

Similarly, abnormal levels of α1-acid glycoprotein (AGP) contribute to the pathogenesis of liver cancer and are a common diagnostic indicator for liver cancer [134]. AGP is synthesized by the liver and is primarily produced by macrophages and liver granulocytes, which are linked to the inflammatory reaction of the body [135]. At present, it has been confirmed that there are five N-glycosylation sites in AGP, which is a complex N-glycan and can form various types of branched conformations of sugar chains, with approximately 11% of the N-glycan chains undergoing sialylation [136]. It has been documented that the glycan conformation of AGP and other modification types on its N-glycan chain are highly correlated with the disease stage, and the level of N-glycan modification of AGP varies among different cancer types [137]. AGP can be used together with other marker proteins for the clinical diagnosis of liver cancer.

Nonglycopeptide-based mass spectrometry (NGP-MS) was used for high throughput screening of serum glycoproteins in patients with liver cancer. Several glycoproteins with potential application value were identified, and their practicability in the diagnosis of liver cancer was evaluated. The results showed that the diagnostic sensitivity of three candidate proteins including AGP was higher than that of AFP, and the combination of them would make the diagnosis more reliable [138].

***Haptoglobin***

Haptoglobin (Hp) is a plasma glycoprotein secreted by the liver, which is generally produced in the acute phase of the response to various stresses [139]. Hp can bind to hemoglobin (Hb), resulting in a new antigenic determinant, which is recognized and cleared by immune cells, thereby maintaining the stability of Hb content and reducing the cytotoxicity produced by Hb, and is an important antagonist of Hb toxicity [140]. Plasma Hp levels sharply rise during an inflammatory condition of the body.

Most serum tumor markers undergo aberrant glycosylation modifications, suggesting that such changes may be useful as a basis for disease diagnosis [141]. For diseases with high mortality rates, such as HCC, reasonable and reliable detection of markers in the early stages of cancer development can reduce false negative results and prevent misclassification to increase patient survival.

Hp is known to have four N-glycosylation sites with various types of sugar chains and mostly sialylation modifications [142]. It has been reported that the N-glycan type of Hp is altered in patients with liver cancer compared with normal adults [143]. Significant differences in the glycosylation levels of serum Hp between patients with cirrhosis and HCC have been reported. Quantitative analysis showed that the abnormal N-glycan structure of Hp occurred at the Asn207 site, as evidenced by the increased level of sialylation and the reduced branching chain structure [144]. Analysis of the structural specificity of glycosylation sites will help in clinical HCC diagnosis.

## Conclusions

N-glycosylation can markedly affect biological processes such as cell adhesion, proliferation, and signal transduction, which in turn are closely related to the hepatocarcinogenesis. Genetic, metabolic, inflammatory responses and the ECM can lead to different degrees of alterations in the N-glycosylation conformation of proteins involved in hepatocarcinogenesis, thus driving the cancer development, including processes such as EMT, ECM changes, and TME formation.

Therefore, targeting N-glycosylation is a promising approach for cancer therapy. Cancer stages have been demonstrated to be closely related to the structural specificity of N-glycosylation, and identifying certain glycosylated epitopes in tumor tissue will aid in patient staging [122]. In addition, much evidence has supported the feasibility of targeting N-glycosylation in immunotherapy [123]. For example, to increase the efficacy of PD-1/PD-L1 immunotherapy, it is necessary to design specific antibodies that can recognize the complex N-glycan structure of PD-L1 itself [124], because the complex glycan structure formed by PD-L1 will obscure its conventional antigen epitopes, affect the binding between PD-L1 and anti-PD-L1 monoclonal antibodies, and increase the possibility of immune escape of cancer cells [125].

Apart from that, protein glycosylation increases molecular heterogeneity and functional diversity within cell populations. As mentioned above, the N-glycan conformation of related proteins is altered during the hepatocarcinogenesis, such as reduced diastereomeric N-glycan structure, abnormal fucosylation, and sialylation. For these aberrant types of N-glycosylation, reducing the aberrant glycosylation phenotype in tumor cells, starting with the relevant glycosyltransferases, may help restore the original cellular activity [126]. However, due to the limitation of technical means, it may also affect the normal expression and function of other N-glycosylated proteins [127].

Understanding the molecular basis behind these N-glycan modifications will help to further comprehend the molecular regulatory mechanisms underlying cancer. With the development of proteomics and other omics technologies, protein N-glycan biosynthesis and its recognition mechanism may become the main drug targets for liver cancer treatment. This innovative strategy is expected to overcome current limitations in the diagnosis, treatment, and prognosis of liver cancer patients.

## Data Availability

All data are available.
